# From Olive Fruits to Olive Oil: Phenolic Compound Transfer in Six Different Olive Cultivars Grown under the Same Agronomical Conditions

**DOI:** 10.3390/ijms17030337

**Published:** 2016-03-04

**Authors:** Nassima Talhaoui, Ana María Gómez-Caravaca, Lorenzo León, Raúl De la Rosa, Alberto Fernández-Gutiérrez, Antonio Segura-Carretero

**Affiliations:** 1 Department of Analytical Chemistry, University of Granada, Avda. Fuentenueva s/n, 18071 Granada, Spain; nassima.talhaoui@gmail.com (N.T.); albertof@ugr.es (A.F.-G.); ansegura@ugr.es (A.S.-C.); 2 Research and Development of Functional Food Centre (CIDAF), PTS Granada, Avda. del Conocimiento s/n, Edificio Bioregión, 18016 Granada, Spain; 3 IFAPA Center of “Alameda del Obispo”, Avda. Menéndez Pidal s/n, E-14004 Córdoba, Spain; lorenzo.leon@juntadeandalucia.es (L.L.); raul.rosa@juntadeandalucia.es (R.D.l.R.)

**Keywords:** phenolic compounds, EVOO, olive fruit, six cultivars, transfer rates

## Abstract

Phenolic compounds are responsible of the nutritional and sensory quality of extra-virgin olive oil (EVOO). The composition of phenolic compounds in EVOO is related to the initial content of phenolic compounds in the olive-fruit tissues and the activity of enzymes acting on these compounds during the industrial process to produce the oil. In this work, the phenolic composition was studied in six major cultivars grown in the same orchard under the same agronomical and environmental conditions in an effort to test the effects of cultivars on phenolic composition in fruits and oils as well as on transfer between matrices. The phenolic fractions were identified and quantified using high-performance liquid chromatography-diode array detector-time-of-flight-mass spectrometry. A total of 33 phenolic compounds were determined in the fruit samples and a total of 20 compounds in their corresponding oils. Qualitative and quantitative differences in phenolic composition were found among cultivars in both matrices, as well as regarding the transfer rate of phenolic compounds from fruits to oil. The results also varied according to the different phenolic groups evaluated, with secoiridoids registering the highest transfer rates from fruits to oils. Moreover, wide-ranging differences have been noticed between cultivars for the transfer rates of secoiridoids (4.36%–65.63% of total transfer rate) and for flavonoids (0.18%–0.67% of total transfer rate). ‘Picual’ was the cultivar that transferred secoiridoids to oil at the highest rate, whereas ‘Changlot Real’ was the cultivar that transferred flavonoids at the highest rates instead. Principal-component analysis confirmed a strong genetic effect on the basis of the phenolic profile both in the olive fruits and in the oils.

## 1. Introduction

In the Mediterranean area, healthy, nutritional, and sensorial properties of olive oil have been known for many centuries. Olive oil is considered the main fat source of the Mediterranean diet, and it is appreciated for its characteristics such as: aroma, taste, color, and nutritive properties that are distinguishable from other vegetable oils. The positive effects of extra-virgin olive oil (EVOO) are likely due not only to the monounsaturated/saturated fatty acid ratio and tocopherols but also to polyphenols. Indeed, many scientific studies have confirmed the healthy benefits of these antioxidant compounds, including the reduction of the risk of coronary disease and of several chronic as well as degenerative diseases such as atherosclerosis, cancer, and strokes [1]. Moreover, polyphenols strongly affect the sensory properties of EVOO such as the typical bitter and pungent taste [2,3], and contribute to the stability of the oil against autoxidation [4].

The amount of polyphenols in EVOO vary, depending on several factors such as geographical zone [5,6,7,8], agro-climatic conditions [9,10,11], degree of fruit ripeness [12] and the oil-extraction process [2,3,13]. Additionally, the phenolic fraction of olive oil can greatly vary among cultivars [6,14], although this aspect has been scarcely studied.

In the olive fruit, the main phenols are secoiridoids such as oleuropein and demethyloleuropein, phenolic glycosides such as ligstroside, and hydroxycinnamic acid derivatives such as verbascoside [15]. During crushing and malaxing processes, oleuropein and demethyloleuropein are hydrolyzed by endogenous β-glycosidases to 3,4-DHPEA-EDA and 3,4-DHPEA-EA. These newly formed substances are the most abundant secoiridoids in olive oil [16]. Jerman Klen *et al.* (2012) [17], studying four cultivars with the same ripening index (RI), demonstrated that during crushing and malaxation in industrial-scale extraction systems, only 0.3%–1.5% of available phenols in olive fruits were transferred to the oil, whereas the rest ended up in wastes. Another study made with one cultivar on a laboratory-scale found that only 0.53% of phenolic compounds ended up in the olive oil [18].

The purpose of the present work was to study the transfer of single phenolic compounds from fruits to oil at the laboratory scale, using six different cultivars grown in the same orchard under the same agronomical and environmental conditions. The results support previous studies related to cultivar effects on phenolic-compound transfer.

## 2. Results and Discussion

### 2.1. Quantitative Characterization of Phenolic Compounds

**Figure 1:**
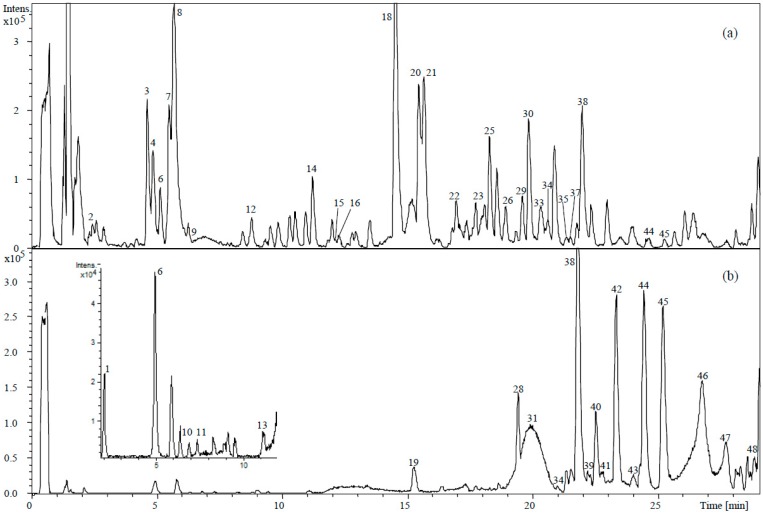
Base peak chromatogram (BPC) of ‘Arbosana’ phenolic compounds of olive fruits (**a**) and olive oil (**b**), using HPLC-DAD-TOF-MS. Proposed phenolic compounds were numbered by elution order (See Table 1 for peak numbers).

The phenolic compounds in olive fruits and oil were identified by the interpretation of their UV-Vis and mass spectra provided by HPLC-DAD-TOF-MS and the information previously reported in the literature. The base-peak chromatograms (BPCs) of two representative phenol extracts of both matrices of the cultivar ‘Arbosana’, in negative ionization mode, are shown in Figure 1. The tentatively identified phenolic compounds are summarized in Table 1, including retention times, *m/z* and molecular formula together with their proposed identities. A total of 33 phenolic compounds were determined in the fruit samples, and a total of 20 compounds were determined in their correspondent oils. Only five compounds (hydroxytyrosol, diosmetin, apigenin, luteolin, and oleuropein aglycone isomer b) were found both in the fruits and in the oil.

Quantification data of olive fruit and oil phenolic compounds for the six cultivars appear in Table 1. As expected, for all cultivars, hydroxytyrosol glucoside and verbascoside were the major phenolic compounds determined in ripe fruits. By contrast, oleuropein aglycone and deacetoxyoleuropein aglycone isomers were the major compounds determined in olive oils. For all cultivars, significant differences were found between the contents of phenolic compounds, both in fruits and in oil. In fact, several papers have reported the genetic effect of the cultivar on the content of phenolic compounds in the fruits as well as in the oil [6,19,20,21]. Overall, the total phenol contents showed low values in fruits as well as in oils, likely due to the late fruit sampling time, as reported in a previous study [22].

‘Changlot Real’ olive fruits showed much higher total phenol content than in the rest of cultivars. Meanwhile the oils of ‘Picual’, ‘Koroneiki’, and ‘Changlot Real’ registered the highest phenol content. This finding highlights the strong effect of the extraction process on olive-oil phenolic content [23].

### 2.2. Transfer of Phenolic Compounds from Fruits to Oil

Phenolic compounds in olive oil underwent marked changes with respect to fruits during oil extraction. These changes, both qualitative and quantitative, were for different reasons. 

#### 2.2.1. Qualitative Changes

In the present study (Table 1) all glycoside phenols were transformed to their aglycone forms, *i.e.*, hydroxytyrosol glucoside, tyrosol glucoside, luteolin glucoside, and apigenin rutinoside. In addition, other complex phenols were completely hydrolyzed to simple phenols, *i.e.*, oleuropein, demethyloleuropein, oleoside, and verbascoside. The complete transformation of those phenols has previously been reported [17]. Such phenol transformation is the result of the activity of many enzymes that are released during pressing and malaxation steps. In particular, polyphenol oxidase could be responsible for an indirect oxidation of secoiridoids, and *β*-glucosidase could play a role in the production of phenol-aglycones such as the deacetoxyoleuropein aglycone, oleuropein aglycone, and their isomers (the main compounds in olive oil) by hydrolysis of oleuropein, dimethyloleuropein, *etc.* [23,24,25,26]. However, some compounds such as ligstroside aglycone and lignans (acetoxypinoresinol and pinoresinol) have curiously been determined in olive oil and not in olive fruit. Ligstroside aglycone is logically the result of ligstroside degradation. It bears noting that ligstroside has previously been detected [22] at early stages of fruit ripening, and then its concentration decreased during ripening to undetectable levels. This could be due to the fact that ligstroside is completely oxidized into other products when fruits ripen, whereas its respected aglycones found in olive oil are the hydrolysis products of other compounds structurally related to ligstroside [18]. In the case of acetoxypinoresinol and pinoresinol, few studies have reported the presence of such lignans in fruits, although most have mentioned the higher amounts of lignans in virgin olive oils than in olive fruits [27,28]. Brenes *et al.* [29] speculated that lignans might originate from the hydrolysis of compounds similar to lignan linked to secoiridoid glucoside. Artajo *et al.* (2007) [23] explained the presence of lignans only in oil by their lipidic character and by the fact that these compounds could be releasable from the vegetable sources after hydrolysis treatments. However, their detection at certain levels in olive stones suggests that lignans in olive oil could come from stones after crushing and malaxation of the whole olive fruits [30].

#### 2.2.2. Quantitative Changes

For both matrices (fruit and olive oil), the total phenolic contents were determined, adding together the individual phenolic contents detected by HPLC-DAD-TOF-MS. For a better understanding of the transference of the individual phenolic compounds from olive fruits to oils, the compounds were grouped into six classes: secoiridoids (oleuropein and isomers, oleuropein glucoside, demethyloleuropein, oleuropein aglycone derivative, oleuropein aglycone and isomers, 10-hydroxyoleuropein aglycone, deacetoxyoleuropein aglycone and isomers, and ligstroside aglycone), flavonoids (luteolin glucoside and isomers, luteolin, apigenin rutinoside, apigenin, rutin, and diosmetin), simple phenols (hydroxytyrosol glucoside and isomers, hydroxytyrosol acetate, hydroxytyrosol, tyrosol glucoside, tyrosol, verbascoside and isomers, *β*-hydroxyverbascoside and isomers, *p*-coumaric acid, and vanillin and isomers), oleosides (6-*p*-coumaroylsecologanoside and isomers, caffeoyl-6-oleoside, and oleoside derivative and isomers), elenolic acid (elenolic acid methyl ester), and lignans (pinoresinol, acetoxypinoresinol).

**Figure 2:**
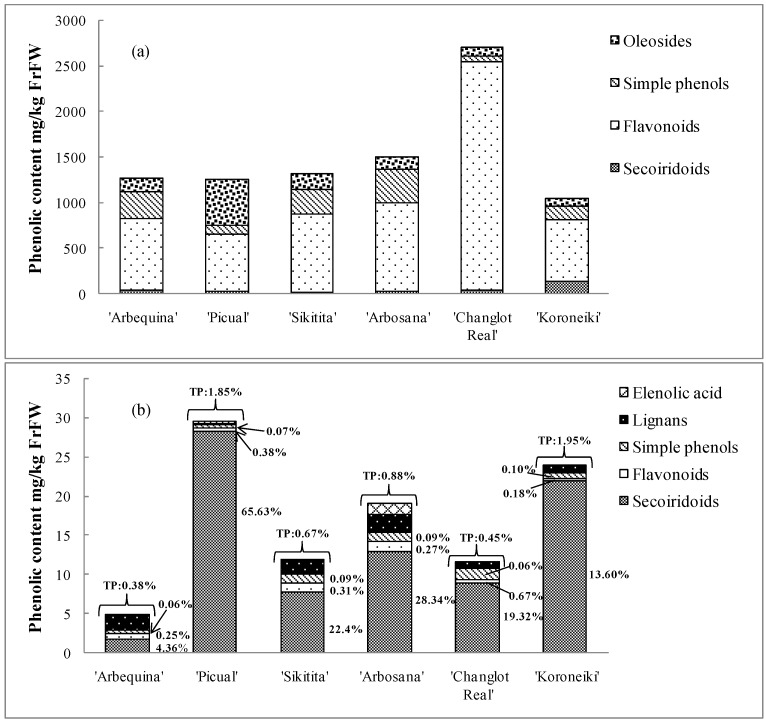
(**a**) Contents of subgroups of phenolic compounds in fruits of various cultivars. (**b**) Contents of subgroups of phenolic compounds in oils produced from the various cultivars. Rates are expressed in % of initial fresh olive fruits phenolic contents (mg/kg fresh fruits weight (FrFW)). TP (total phenol).

In Figure 2a,b, the groups and total phenolic contents are expressed with the same unit mg/kg of fruit fresh weight (FrFW) for both matrices (fruits and olive oil). Figure 2b also presents the phenol transfer rates between fruits and oil of the six different cultivars. Those rates have been calculated and expressed as a percentage of initial phenolic content of fresh olive fruits taking into account the percentage of olive oil produced from one kilogram of olive fruits. In general terms, a very low percentage of total phenols were transferred from fruits to oils for all cultivars (0.38%–1.95%). The result agrees with a previous report where only 0.3%–1.5% of available phenols were transferred to olive oil, whereas the rest ended up in wastes (>40%), depending on the extraction process [17]. In the present study, the total phenol transfer rate varied markedly among cultivars, although the same abencor-system extraction was used to produce the oil for all cultivars. Thus, the hypothesis of the effect of the extraction process could be discarded. Notably, cultivars with lower phenol-transfer rates coincided with those that presented a high percentage of moisture in fruit and vice versa (moisture data not shown); that is, ‘Arbequina’ and ‘Changlot Real’ presented the lowest transfer rates (0.38% and 0.45%) simultaneously with the highest fruit moisture (67%–72%), whereas ‘Picual’ and ‘Koroneiki’ showed the highest transfer rates (1.85% and 1.95%) and the lowest fruit moisture (62% for both). Olive-oil phenolic compounds are amphiphilic in nature and are more soluble in the water than in the oil phase [31]. In addition, during oil extraction from olives, phenolic compounds are distributed between the oil and aqueous phases [32]. Therefore, and because all the samples analyzed received the same irrigation and precipitation, it can be conjectured that the fruit moisture of each cultivar negatively affects the transfer of phenolic compounds to the oil. Because water uptake is cultivar dependent [33], this result could highlight the influence of the genetic factor in the transfer of phenolic compounds from olive fruit to oil.

Among all phenolic groups, secoiridoids were the compounds with the highest transfer rate from fruits to oil, followed by flavonoids and simple phenols (Figure 2b). In fact, secoiridoids, which are the most lipophilic compounds, may have undergone semi-degradation during crushing and malaxation, but this large phenolic group was still present in the oil in their aglycone forms. The dominance of secoiridoid derivatives, followed by flavonoids and phenolic alcohols in oil have also been reported by Artajo *et al*. (2007) [23] and Jerman Klen *et al.* (2015) [18]. By contrast, the low transfer of flavonoids is presumably due to the fact that rutin, the major flavonoid found in olive fruits, is completely wasted in water without many alterations, such as hydrolysis and/or other degradation reactions, during the oil process [17,23,34]. The oleoside groups completely disappeared in oil, perhaps due to their above-mentioned degradation pathways to simple phenols. On the contrary, a new group of lignans appeared in the oil, and the appearance of this group was explained above for pinoresinol and acetoxypinoresinol. 

Furthermore, no clear differences were detected in the transfer rates of simple phenols among cultivars (0.06%–0.10%). However, sharp differences were registered for secoiridoids (4.36%–65.63%) and for flavonoids (0.18%–0.67%) in terms of transfer rates. In fact, secoiridoids had high transfer rates in ‘Picual’ cultivar, as did flavonoids in ‘Changlot Real’ cultivar. This trend was not correlated with the original contents of secoiridoids and flavonoids in fruits of those cultivars, contradicting the deduction that more phenols in fruits would lead automatically to more phenols in oil. The result found is probably due to the amphiphilic character of those phenolic groups in interaction with the humidity of each cultivar.

### 2.3. Chemometric Analysis 

**Figure 3:**
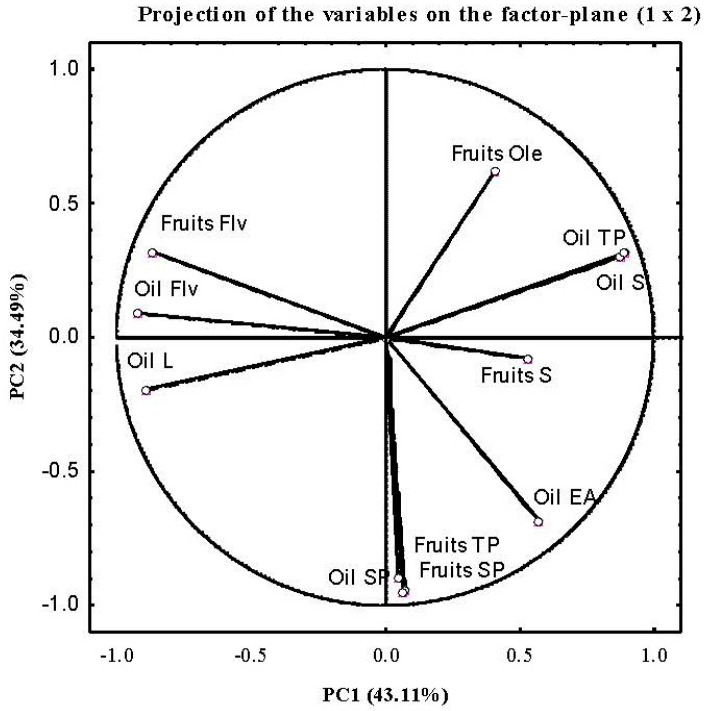
Projection on the factorial plane of olive and oils variables. Ole (oleosides), TP (total phenols), S (secoiridoids), EA (elenolic acid), SP (simple phenol), Flv (flavonoid) and L (lignans).

**Figure 4:**
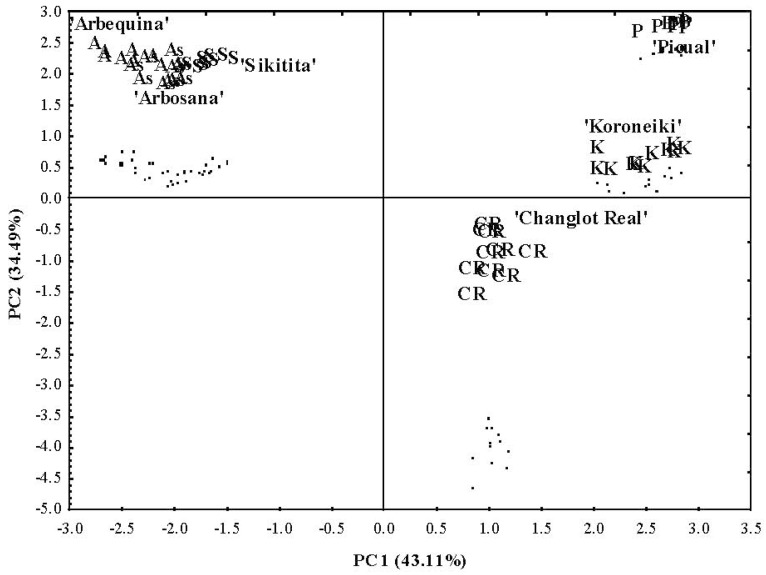
Scatter plots for the first and second principal components for the different cultivars. P (‘Picual’), K (‘Koroneiki’), CR (‘Changlot Real’), S (‘Sikitita’), As (‘Arbosana’) and A (‘Arbequina’).

PCA was applied to the contents of different phenolic groups’ and to total phenols of olive fruits as well as oils at the same time. The first (PC1) and second (PC2) principal components described more than 77.60% of the data variability for all cases of the analysis. PC1 was clearly linked to fruits and oil secoiridoids, fruit and oil flavonoids, oil lignans, and oil total phenols, whereas PC2 was correlated to fruit and oil simple phenols, fruits oleosides, and total fruit phenols (Figure 3). Notably, the chemometric analysis showed that phenolic groups and total phenolic contents of fruits and oils were responsible for the discrimination of almost all cultivars (Figure 4). In fact, the different cultivars were greatly separated, except for ‘Arbequina’, ‘Sikitita’, and ‘Arbosana’. This result again confirms the high genetic variability in the phenolic compound profiles in olive fruits [35,36] as well as oil [37,38]. The difficulty of distinguishing the cultivars ‘Arbequina’, ‘Sikitita’ and ‘Arbosana’ is no doubt due to the proximity of their phenolic profiles. In fact, ‘Arbequina’ and ‘Sikitita’ are genetically related (‘Sikitita’ comes from a cross between ‘Picual’ × ‘Arbequina’ [39]), and a higher degree of similarity of ‘Sikitita’ oil phenol composition with the ‘Arbequina’ than with the ‘Picual’ parent has previously been reported [40]. ‘Arbequina’ and ‘Arbosana’ originated from the same geographical area (Catalonia, Spain) and thus could also be genetically related [41].

## 3. Materials and Methods

### 3.1. Chemicals and Reagents

Standard compounds such as hydroxytyrosol, tyrosol, luteolin, apigenin, and pinoresinol were purchased from Sigma-Aldrich (St. Louis, MO, USA), and oleuropein from Extrasynthèse (Lyon, France). Methanol reagent was from Panreac (Barcelona, Spain). HPLC-grade acetonitrile and acetic acid (assayed at >99.5%) used for preparing mobile phases were from Labscan (Dublin, Ireland) and Fluka (Switzerland), respectively. Distilled water with a resistance of 18.2 MΩ was deionized in a Milli-Q system (Millipore, Bedford, MA, USA). The stock solutions containing these analytes were prepared in methanol. All chemicals were of analytical reagent grade and used as received. All the solutions were stored in dark flasks at −20 °C until used.

### 3.2. Samples

Olives from the cultivars ‘Arbosana’, ‘Koroneiki’, ‘Picual’, ‘Sikitita’, ‘Arbequina’, and ‘Changlot Real’ were harvested at the same time in mid-December from the same olive orchards in “IFAPA, Centro Alameda del Obispo” in Córdoba, Spain (37°51'36.5"N 4°47'53.7"W). These cultivars were selected as some of the most widely used in new orchards currently cultivated in Spain, highly productive, and well adapted to modern olive-growing techniques. Only healthy fruits without any kind of disease or physical damage were processed. Olive-oil samples were prepared at the laboratory scale using the Abencor system (Comercial Abengoa, S.A., Seville, Spain) equipped with a hammer crusher, malaxer, and centrifuge that simulates the industrial process of EVOO production. Malaxation was carried out at 25 °C for 30 min and centrifugation of the kneaded paste was performed in a basket centrifuge at 3500 rpm for 1 min. After centrifugation, the oils were decanted, paper filtered, and transferred into dark glass bottles until analysis.

### 3.3. Extraction of Phenolic Compounds from Olive Fruits and Oils

First, 2 g of fresh olive fruits (FrF) were crushed and extracted via Ultra-Turrax IKA T18 basic with 30 mL of MeOH/H_2_O (80/20), and afterwards the sample was placed in an ultrasonic bath (10 min) and centrifuged at 4500 rpm for 15 min. Next, the supernatant was removed, and the extraction was repeated twice more. The supernatants were collected and the extract was then evaporated. Then, the extract was reconstituted with 20 mL of acidified water (at pH 2.3) and washed up twice with 40 mL of hexane to remove the possible oil. Then 40 mL of methanol was added to the aqueous solution and evaporated again. Finally, the extract was reconstituted with 2 mL of MeOH/H_2_O (50/50). 

The polar fraction of olive oil was extracted as described elsewhere [7] with some modifications. Briefly, 2 g of oil sample were weighed and washed with 3 mL of hexane. Afterwards, 2 mL of methanol: water (60/40) were added, the mixture was vortexed and then centrifuged at 3000 rpm for 5 min. Then, the supernatant was removed, and the extraction was repeated twice more. The polar extract was evaporated in a rotary evaporator. The residue was dissolved in 0.25 mL of MeOH/H_2_O (50/50).

### 3.4. Determination of Phenolic Compounds by HPLC-DAD-TOF-MS

HPLC analyses were carried out using an Agilent 1200 series Rapid Resolution Liquid Chromatograph (Agilent Technologies, Santa Clara, CA, USA). The phenolic fractions were separated in a Poroshell 120 EC-C18 analytical column (4.6 mm × 100 mm, 2.7 μm). The gradient eluent was used at flow rate of 0.8 mL/min, following the method described by Talhaoui *et al.* [42]. The column temperature was maintained at 25 °C and the injection volume was 2.5 μL.

In addition, the HPLC system was coupled to a micrOTOF (Bruker Daltonics, Bremen, Germany), an orthogonal-accelerated TOF mass spectrometer, using an electrospray interface (model G1607A from Agilent Technologies, Palo Alto, CA, USA). The effluent from the HPLC column was split using a T-type phase separator before being introduced into the mass spectrometer (split ratio = 1:3). Analysis parameters were set using a negative-ion mode with spectra acquired over a mass range from *m/z* 50 to 1000. The optimum values of the ESI-MS parameters were: capillary voltage, +4.5 kV; drying gas temperature, 190 °C; drying gas flow, 9.0 L/min; and nebulizing gas pressure, 2 bars. The accurate mass data on the molecular ions was processed through Data Analysis 4.0 software (Bruker Daltonics, Bremen, Germany), which provided a list of possible elemental formulae via the Smart Formula Editor. The Smart Formula Editor uses a CHNO algorithm, which provides standard functionalities such as minimum/maximum elemental range, electron configuration and ring-plus double-bond equivalents, as well as a sophisticated comparison of the theoretical with the measured isotope pattern (Sigma Value) for increased confidence in the suggested molecular formula. The quantification was carried out using Bruker Compass Target Analysis 1.2 software for compound screening (Bruker Daltonics, Bremen, Germany). 

Quantification was made according to the linear calibration curves of standard compounds. Different calibration curves were prepared using the following standards: oleuropein, hydroxytyrosol, tyrosol, apigenin, luteolin, and pinoresinol. All calibration curves showed good linearity among different concentrations. The calibration plots revealed good correlation between peak areas and analyte concentrations, and the regression coefficients in all cases were higher than 0.995. Limit of detection (LOD) was found to be within the range 0.053–0.233 µg/mL whereas the limit of quantification (LOQ) was within 0.175–0.679 µg/mL.

### 3.5. Statistical Analysis

All the statistical analyses (ANOVA and principal-component analysis) were performed using Statistica 8.0 software (2001, StatSoft, Tulsa, OK, USA). Samples were collected from three trees per cultivar and all assays were run in triplicate. Significant statistical differences among treatments (*p* < 0.01) were assessed by Tukey’s honest significant-difference multiple comparisons. Values of different results of phenolic compounds were expressed as the means mg/kg fresh fruits weight (FrFW), and as the means mg/kg olive oil. The principal-components analysis (PCA) was performed to detect structure in the relationships between variables, allowing the classification and the separation of each cultivar. 

## 4. Conclusions

In summary, phenolic compounds displayed sharp qualitative and quantitative differences among the cultivars considered in the present study and among olive fruits and olive oils. Specifically, after fruit processing, new compounds appeared in oil, notably aglycone forms because of the partial or total degradation during oil process of some original compounds detected in fruits, or totally new structures such as lignans. The phenolic transfer rate did not surpass 2% in all cultivars; however, pronounced differences among cultivars in transfer rates were detected in total phenol and individual phenolic groups. These results clearly reveal the genetic contribution to olive phenolic content and composition as well as their transfer between olive fruits and oil.

## Figures and Tables

**Table 1:** Phenolic compounds determined in olive fruits and oils extract by HPLC-DAD-TOF-MS, including retention time, *m/z*, formula, means of compounds by cultivar, and total (mg/kg of fruit fresh weight (FrFW) or oil). Standard deviations (in parentheses); n.q. (not quantified); and n.i. (not identified).

Compounds *^a^*		Rt min	*m/z*	Formula	Phenolic Content (mg/kg FrFW or mg/kg oil)											
					‘Arbequina’ Fruit Oil		‘Picual’ Fruit Oil		‘Sikitita’ Fruit Oil		‘Arbosana’ Fruit Oil		‘Changlot Real’ Fruit Oil		‘Koroneiki’ Fruit Oil	
1	Vanillin isomer a ^3^	2.09	151	C_8_H_8_O_3_	n.i.	0.10 (0.01)	n.i.	0.18 (0.01)	n.i.	0.21 (0.02)	n.i.	0.40 (0.01)	n.i.	0.11 (0.01)	n.i.	0.12 (0.01)
2	Hydroxytyrosol glucoside isomer a ^3^	2.31	315	C_14_H_20_O_8_	n.i.	n.i.	n.i.	n.i.	n.i.	n.i.	n.i.	n.i.	91.74 (7.53)	n.i.	n.i.	n.i.
3	Hydroxytyrosol glucoside isomer b ^3^	4.58	315	C_14_H_20_O_8_	n.i.	n.i.	n.i.	n.i.	212.34 (23.07)	n.i.	n.i.	n.i.	n.i.	n.i.	n.i.	n.i.
4	Hydroxytyrosol glucoside isomer c ^3^	4.8	315	C_14_H_20_O_8_	n.i.	n.i.	79.68 (7.89)	n.i.	56.37 (2.04)	n.i.	n.i.	n.i.	440.58 (40.67)	n.i.	96.80 (7.09)	n.i.
5	Hydroxytyrosol glucoside isomer d ^3^	4.82	315	C_14_H_20_O_8_	257.39 (22.06)	n.i.	58.32 (5.01)	n.i.	n.i.	n.i.	276.71 (16.71)	n.i.	433.30 (35.44)	n.i.	114.74 (7.39)	n.i.
6	Hydroxytyrosol ^3^	5.14	153	C_8_H_10_O_3_	62.91 (4.93)	0.29 (0.01)	107.97 (7.40)	1.12 (0.04)	85.16 (8.26)	0.83 (0.07)	73.41 (6.08)	1.39 (0.07)	73.53 (7.59)	1.63 (0.713)	61.61 (5.43)	1.57 (0.11)
7	Oleoside derivative isomer a ^4^	5.67	407	C_17_H_28_O_11_	20.22 (1.17)	n.i.	12.26 (0.98)	n.i.	19.80 (0.76)	n.i.	24.33 (1.18)	n.i.	23.36 (1.95)	n.i.	17.67 (1.87)	n.i.
8	Oleoside derivative isomer b ^4^	6.26	407	C_17_H_28_O_11_	57.93 (3.00)	n.i.	231.21 (22.43)	n.i.	72.07 (3.41)	n.i.	71.31 (6.25)	n.i.	63.80 (4.94)	n.i.	52.48 (4.32)	n.i.
9	Tyrosol glucoside ^3^	6.59	299	C_14_H_20_O_7_	n.i.	n.i.	n.i.	n.i.	46.27 (3.88)	n.i.	n.i.	n.i.	615.18 (49.57)	n.i.	n.i.	n.i.
10	Vanillin isomer b ^3^	6.81	151	C_8_H_8_O_3_	n.i.	0.022 (0.001)	n.i.	n.i.	n.i.	0.040 (0.004)	n.i.	0.028 (0.002)	n.i.	n.i.	n.i.	n.i.
11	Tyrosol ^3^	7.28	137	C_8_H_10_O_2_	n.i.	n.i.	n.i.	1.99 (0.12)	n.i.	2.28 (0.24)	n.i.	2.06 (0.15)	n.i.	10.25 (0.96)	n.i.	3.46 (0.12)
12	*p*-coumaric acid ^3^	8.73	163	C_8_H_8_O_3_	27.88 (2.35)	n.i.	27.51 (2.40)	n.i.	61.71 (4.57)	-	79.01 (5.59)	n.i.	21.09 (2.03)	n.i.	53.50 (3.19)	n.i.
13	Vanillin isomer c ^3^	11.06	151	C_8_H_8_O_3_	n.i.	0.27 (0.03)	n.i.	n.i.	n.i.	0.18 (0.02)	n.i.	0.28 (0.02)	n.i.	n.i.	n.i.	n.i.
14	Oleuropein aglycone derivative ^1^	11.44	377	C_16_H_26_O_10_	12.70 (0.42)	n.i.	18.47 (1.12)	n.i.	11.72 (0.87)	-	21.01 (1.80)	-	24.55 (2.38)	n.i.	106.25 (9.67)	n.i.
15	*β*-hydroxy-verbascoside isomer a ^3^	12.06	639	C_29_H_36_O_16_	n.i.	n.i.	5.22 (0.08)	n.i.	11.40 (0.72)	-	10.99 (0.54)	-	16.96 (1.65)	n.i.	5.99 (0.42)	n.i.
16	*β*-hydroxy-verbascoside isomer b ^3^	12.21	639	C_29_H_36_O_16_	n.i.	n.i.	2.36 (0.07)	n.i.	10.19 (0.79)	-	14.02 (1.32)	-	15.07 (0.77)	n.i.	4.02 (0.40)	n.i.
17	Demethyloleuropein ^1^	13.95	525	C_24_H_30_O_13_	9.75 (0.94)	n.i.	23.68 (2.15)	n.i.	n.i.	n.i.	n.i.	n.i.	n.i.	n.i.	n.i.	n.i.
18	Rutin ^2^	14.48	609	C_27_H_30_O_16_	110.16 (6.09)	n.i.	19.48 (1.79)	n.i.	114.34 (10.88)	-	189.44 (18.01)	-	20.27 (0.45)	n.i.	113.53 (10.67)	n.i.
19	Hydroxytyrosol acetate/3,4-DHPEA-AC ^3^	15.22	195	C_10_H_12_O_4_	n.i.	2.67 (0.20)	n.i.	n.i.	n.i.	2.06 (0.14)	n.i.	2.37 (0.08)	n.i.	n.i.	n.i.	n.i.
20	Luteolin glucoside isomer ^2^	15.4	447	C_21_H_20_O_11_	149.29 (10.75)	n.i.	18.87 (0.70)	n.i.	69.84 (3.29)	n.i.	129.79 (12.63)	n.i.	24.94 (2.42)	n.i.	14.92 (0.74)	n.i.
21	Verbascoside isomer a ^3^	15.61	623	C_29_H_36_O_15_	340.74 (33.43)	n.i.	307.91 (25.61)	n.i.	308.55 (13.75)	n.i.	406.40 (28.24)	n.i.	731.26 (59.36)	n.i.	292.94 (27.97)	n.i.
22	Verbascoside isomer b ^3^	16.96	623	C_29_H_36_O_15_	103.94 (8.40)	n.i.	37.58 (3.75)	n.i.	69.32 (4.01)	n.i.	101.78 (8.34)	n.i.	58.20 (4.96)	n.i.	46.35 (4.59)	n.i.
23	Apigenin rutinoside ^2^	17.95	577	C_27_H_30_O_14_	5.94 (0.39)	n.i.	4.32 (0.41)	n.i.	6.14 (0.50)	n.i.	7.81 (0.70)	n.i.	n.i.	n.i.	2.12 (0.23)	n.i.
24	Oleuropein glucoside ^1^	18.05	701	C_31_H_42_O_18_	n.i.	n.i.	n.i.	n.i.	n.i.	n.i.	24.98 (2.43)	n.i.	3.50 (0.30)	n.i.	n.i.	n.i.
25	Caffeoyl-6-oleoside ^4^	18.48	551	C_25_H_28_O_14_	n.i.	n.i.	87.76 (8.34)	n.i.	24.98 (2.42)	n.i.	n.i.	n.i.	n.i.	n.i.	n.i.	n.i.
26	Oleuropein isomer a ^1^	18.87	539	C_25_H_32_O_13_	n.i.	n.i.	n.i.	n.i.	n.i.	n.i.	7.82 (0.77)	n.i.	n.i.	n.i.	n.i.	n.i.
27	Oleuropein isomer b ^1^	19.07	539	C_25_H_32_O_13_	n.i.	n.i.	3.93 (0.28)	n.i.	n.i.	n.i.	4.38 (0.23)	n.i.	5.47 (0.59)	n.i.	n.i.	n.i.
28	10-Hydroxyoleuropein aglycone ^1^	19.38	335	C_17_H_20_O_7_	n.i.	0.71 (0.05)	-	0.62 (0.04)	n.i.	7.91 (0.86)	n.i.	3.20 (0.27)	n.i.	0.23 (0.02)	n.i.	0.22 (0.02)
29	Oleuropein isomer c ^1^	19.53	539	C_25_H_32_O_13_	1.80 (0.09)	n.i.	1.46 (0.14)	n.i.	n.i.	n.i.	n.i.	n.i.	5.37 (0.18)	n.i.	29.05 (2.21)	n.i.
30	6-*p*-Coumaroyl secologanoside isomer a ^4^	19.80	535	C_25_H_28_O_13_	58.93 (1.82)	n.i.	176.42 (13.78)	n.i.	50.38 (4.10)	n.i.	45.13 (3.61)	n.i.	9.89 (0.84)	n.i.	14.29 (1.39)	n.i.
31	Deacetoxyoleuropein aglycone isomer a ^1^	19.87	319	C_17_H_20_O_6_	n.i.	7.77 (0.62)	n.i.	1.26 (0.09)	n.i.	12.14 (1.20)	n.i.	29.86 (2.29)	n.i.	2.14 (0.22)	n.i.	2.90 (0.09)
32	Oleuropein isomer d ^1^	20.15	539	C_25_H_32_O_13_	n.i.	n.i.	n.i.	n.i.	2.32 (0.17)	n.i.	n.i.	n.i.	n.i.	n.i.	n.i.	n.i.
33	Oleuropein isomer e ^1^	20.47	539	C_25_H_32_O_13_	4.18 (0.17)	n.i.	n.i.	n.i.	n.i.	n.i.	n.i.	n.i.	n.i.	n.i.	n.i.	n.i.
34	Oleuropein aglycone isomer a ^1^	20.59	377	C_19_H_22_O_8_	n.i.	n.i.	4.29 (0.22)	n.i.	n.i.	n.i.	n.i.	n.i.	9.52 (0.87)	n.i.	n.i.	12.22 (1.01)
35	6-*p*-Coumaroyl secologanoside isomer b ^4^	20.72	535	C_25_H_28_O_13_	2.63 (0.26)	n.i.	1.43 (0.11)	n.i.	n.i.	n.i.	2.07 (0.19)	n.i.	n.i.	n.i.	n.i.	n.i.
36	Oleuropein aglycone isomer b ^1^	20.86	377	C_19_H_22_O_8_	n.i.	n.i.	9.89 (0.82)	n.i.	n.i.	n.i.	n.i.	n.i.	n.i.	n.i.	n.i.	n.i.
37	Oleuropein isomer f ^1^	21.22	539	C_25_H_32_O_13_	n.i.	n.i.	3.86 (0.29)	n.i.	n.i.	n.i.	n.i.	n.i.	n.i.	n.i.	n.i.	n.i.
38	Luteolin ^2^	21.94	285	C_15_H_10_O_6_	25.42 (1.93)	3.51 (0.31)	47.50 (2.81)	1.93 (0.08)	68.53 (6.57)	3.19 (0.20)	33.01 (3.24)	3.65 (0.38)	18.60 (1.34)	2.20 (0.04)	18.44 (1.01)	1.31 (0.08)
39	Deacetoxyoleuropein aglycone isomer b ^1^	22.29	319	C_17_H_20_O_6_	n.i.	n.q.	n.i.	1.09 (0.09)	n.i.	0.11 (0.01)	n.i.	0.71 (0.03)	n.i.	0.05 (0.01)	n.i.	n.q.
40	Oleuropein aglycone c ^1^	22.48	377	C_19_H_22_O_8_	n.i.	0.68 (0.06)	n.i.	1.76 (0.10)	n.i.	1.02 (0.07)	n.i.	0.32 (0.03)	n.i.	0.94 (0.08)	n.i.	1.98 (0.15)
41	Elenolic acid methyl ester ^5^	22.61	255	C_12_H_16_O_6_	n.i.	0.18 (0.01)	n.i.	0.21 (0.02)	n.i.	0.12 (0.01)	n.i.	1.55 (0.15)	n.i.	n.i.	n.i.	-
42	Acetoxypinoresinol ^6^	23.3	415	C_22_H_24_O_8_	n.i.	13.04 (1.39)	n.i.	0.13 (0.01)	n.i.	8.27 (0.80)	n.i.	11.70 (0.76)	n.i.	7.00 (0.47)	n.i.	5.88 (0.55)
43	Pinoresinol ^6^	23.93	357	C_20_H_22_O_6_	n.i.	0.46 (0.04)	n.i.	n.i.	n.i.	0.42 (0.03)	n.i.	0.81 (0.08)	n.i.	n.i.	n.i.	n.i.
44	Apigenin ^2^	24.62	269	C_15_H_10_O_5_	0.49 (0.03)	1.42 (0.04)	1.52 (0.09)	0.73 (0.02)	1.69 (0.17)	1.06 (0.06)	8.22 (0.92)	3.73 (0.21)	1.38 (0.04)	1.15 (0.02)	1.09 (0.07)	0.72 (0.05)
45	Diosmetin ^2^	25.54	299	C_16_H_12_O_6_	0.53 (0.05)	0.55 (0.03)	n.i.	n.q.	2.64 (0.26)	1.91 (0.08)	n.i.	0.12 (0.01)	n.i.	n.q.	n.i.	n.q.
46	Oleuropein aglycone d ^1^	26.73	377	C_19_H_22_O_8_	n.i.	2.74 (0.19)	n.i.	89.63 (7.49)	n.i.	17.49 (1.76)	n.i.	21.46 (2.92)	n.i.	57.21 (4.83)	n.i.	118.39 (16.09)
47	Oleuropein aglycone c ^1^	27.79	377	C_19_H_22_O_8_	n.i.	0.96 (0.07)	n.i.	36.13 (3.41)	n.i.	6.62 (0.35)	n.i.	14.28 (1.11)	n.i.	4.49 (0.22)	n.i.	3.43 (0.30)
48	Ligstroside aglycone ^1^	28.76	361	C_19_H_22_O_7_	n.i.	0.54 (0.05)	n.i.	32.08 (2.22)	n.i.	0.45 (0.05)	n.i.	1.20 (0.08)	n.i.	3.72 (0.17)	n.i.	3.56 (0.20)
	**Total**				**1265.33 (55.34)**	**35.92 (1.68)**	**1249.35 (53.14)**	**173.13 (8.66)**	**1318.03 (48.95)**	**66.33 (2.16)**	**1508.95 (43.54)**	**99.20 (4.55)**	**2699.89 (200.56)**	**155.76 (16.69)**	**1066.84 (47.69)**	**169.56 (7.65)**

*^a^* Superscript numbers indicate phenolic groups: ^1^, secoiridoids; ^2^, flavonoids; ^3^, simple phenols; ^4^, oleosides; ^5^, elenolic acids; ^6^, lignans.
